# Obesity Prevalence and Its Impact on Maternal and Neonatal Outcomes in Pregnant Women: A Systematic Review

**DOI:** 10.7759/cureus.75262

**Published:** 2024-12-07

**Authors:** Fai S Almutairi, Alaa M Alsaykhan, Abrar A Almatrood

**Affiliations:** 1 Obstetrics and Gynecology, Maternity and Children Hospital, Buraydah, SAU; 2 Obstetrics and Gynecology, Maternity and Children Hospital, Hail, SAU

**Keywords:** maternal, neonatal, obesity, outcome, pregnancy

## Abstract

Globally, obesity prevalence has progressively increased and is now at epidemic levels; this trend is mirrored in women of childbearing age. There is a high level of evidence that maternal obesity is associated with a range of adverse pregnancy complications and neonatal outcomes, such as hypertensive disorders of pregnancy, gestational diabetes mellitus (GDM), large for gestational age (LGA) fetuses, premature birth, stillbirth, cesarean section, and postpartum hemorrhage, among certain others. This systematic review aimed to comprehensively evaluate the relationship between maternal obesity and health outcomes for both mothers and infants. The inclusion criteria encompass studies focusing on pregnant women with obesity, research examining obesity prevalence in pregnancy, and investigations into various maternal and neonatal outcomes. Quality assessment was performed using the Newcastle-Ottawa Scale to ensure the reliability and validity of findings, while meta-analysis was performed to calculate the pooled prevalence of obesity. The findings highlight significant associations between maternal obesity and adverse outcomes for both mothers and neonates, respectively. Increased gestational weight gain in obese individuals correlates with a higher risk of complications, such as cesarean delivery, preeclampsia, and postpartum hemorrhage. Specifically, obesity has been consistently linked to higher rates of GDM, which further elevates the likelihood of cesarean sections and other complications during labor. Additionally, in terms of neonatal outcomes, studies reveal that maternal obesity influences the incidence of LGA infants, often leading to macrosomia. Neonates born to obese mothers may also have increased rates of NICU admissions, reflecting the challenges posed by higher maternal weight and its associated risks. Maternal obesity is consistently associated with adverse maternal and neonatal outcomes. However, diversity in outcomes, such as Apgar scores, underscores the need for further research to better understand these complex relationships.

## Introduction and background

Obesity has been recognized as a major public health concern, ranking sixth among the leading causes of mortality worldwide. Overweight and obesity are two of the most prevalent lifestyle diseases, which lead to additional medical conditions and contribute to an array of chronic diseases, including cancer, diabetes, metabolic syndrome, and cardiovascular disease. The World Health Organization also anticipated that by 2030, 30% of deaths worldwide will be caused by lifestyle diseases [1]. Obesity prevalence is highest among women of reproductive age post-adolescent and pre-menopausal, with an average annual weight gain of 0.5-1 kg from early adulthood to mid-life. Critically, it indicates that even young women are at risk of harmful weight increases as they approach childbearing age. This has a substantial impact on women's reproductive health because they start pregnancy at a higher BMI. Obese women with a BMI ≥40 kg/m^2^ have a nearly seven-fold increased risk of waiting more than 12 months to conceive compared to those with a normal BMI. Women who are overweight or obese are also more likely to have pregnancy issues such as gestational diabetes mellitus (GDM), gestational hypertension, and preeclampsia. In addition to being overweight or obese before and during pregnancy, the amount of weight gained by women during pregnancy, referred to as gestational weight gain (GWG), is critical for favorable maternal and neonatal outcomes. Women who were obese before pregnancy are more likely to have a higher GWG than normal-weight women. However, excess GWG is an independent risk factor for a myriad of complications during pregnancy, exposing women to risk for future cardiometabolic diseases [2].

Previous studies demonstrated an odds ratio (OR) of 1.1 for severe maternal morbidity in women with obesity class 1 (BMI=30.0-34.9) versus those with normal weight (BMI=18.5-24.9). The OR for obesity class 2 (BMI=35.0-39.8) is 1.2, whereas for obesity class 3 (BMI≥40) is 1.4. Maternal obesity increases perinatal mortality, making obesity the most prevalent health concern among women of reproductive age [3]. Obesity during pregnancy has both acute and long-term negative repercussions for the mother and child. Obesity causes infertility and, during early gestation, spontaneous pregnancy loss and congenital abnormalities. Obese women have higher insulin resistance in early pregnancy, which shows up clinically in late gestation as glucose intolerance and fetal overgrowth. At term, there is a higher chance of cesarean delivery and wound complications [4]. Obese postpartum women are more likely to experience venous thromboembolism, depression, and trouble breastfeeding. Because 50-60% of overweight or obese women gain more than gestational weight guidelines, postpartum weight retention raises future cardiometabolic risks and prepregnancy obesity in successive pregnancies. Neonates of obese women have higher body fat levels at birth, which increases the likelihood of juvenile obesity. Although there is no single mechanism that accounts for the negative perinatal outcomes associated with maternal obesity, the evidence at hand suggests that increased prepregnancy maternal insulin resistance and associated hyperinsulinemia, inflammation, and oxidative stress contribute to early placental and fetal dysfunction [4].

Obesity rates among pregnant women increased from 11.1% in 2012 to 13.4% in 2018, with age-related weight gain. Pregnant women from ethnic groups had a significant incidence of overweight and obesity as follows: the ORs for Maghrebi, Sub-Saharan African, and Latin American populations were 4.08, 3.18, and 1.59, respectively [5]. The incidence of overweight and obesity varies greatly around the world, with women having the highest prevalence rates in the Pacific island nations, the Caribbean, and the Middle East. Globally, it is estimated that up to 39 million pregnancies are exacerbated by maternal obesity each year [3,6], and in some countries, the estimated prevalence of overweight and obesity during pregnancy exceeds 60-64% in South Africa, 65% in Mexico, and 55-63% in the United States [3,6,7]. In England, the incidence of overweight and obesity is 35% among 16-24 years old women and 61% among 35-44 years old women, indicating a substantial risk for women of reproductive age [6,8]. Antenatal obesity is particularly prevalent in low-income areas, in older mothers, and among minority ethnic groups [6].

Over the last three decades, the prevalence and increase of overweight and obese status have increased substantially in both the general population and pregnant women. This increase is shown in both higher pre-pregnancy BMI measures and excessive weight gain during pregnancy. Maternal obesity has been demonstrated to worsen comorbidities such as insulin resistance, pregnancy-induced hypertension, and infectious diseases in parturient mothers. These alterations have been demonstrated to increase the rate of fetal abnormalities and affect fetal growth, as well as other elements of delivery, such as instrumented vaginal deliveries and an increase in cesarean section deliveries. Maternal obesity raises fetal birth weight and affects neonatal resuscitation by increasing the need for respiratory support [9]. Maternal obesity alone increases the risk of poor newborn outcomes such as macrosomia, perinatal mortality, induced preterm birth, and birth anomalies. Excess prenatal weight gain, particularly early in pregnancy, raises the odds of juvenile obesity and higher fat mass [10].

This systematic review aimed to comprehensively evaluate the relationship between maternal obesity and health outcomes for both mothers and infants. Obesity in pregnancy is known to increase the risk of various complications for mothers. Additionally, it has significant implications for neonatal health, including higher rates of stillbirth, preterm birth, macrosomia, and diverse others. By systematically reviewing and synthesizing existing evidence from multiple studies, this review seeks to provide a clear understanding of the prevalence of obesity among pregnant women and its specific impacts on maternal and neonatal health outcomes. This synthesis is crucial for informing clinical practice guidelines and public health strategies aimed at mitigating the adverse effects of maternal obesity, ultimately improving outcomes for both mothers and their babies.

## Review

Material and methods

Definition of Outcomes and Inclusion Criteria

This study aimed to determine the prevalence of obesity among pregnant women and evaluate its impact on both maternal and neonatal outcomes. The inclusion criteria encompass studies focusing on pregnant women with obesity, research examining obesity prevalence in pregnancy, and investigations into various maternal outcomes such as gestational diabetes, postpartum hemorrhage, preeclampsia, cesarean section rates, and other related complications. Additionally, included studies assess neonatal outcomes associated with maternal obesity; including preterm birth; birth weight; appearance, pulse, grimace, activity, and respiration (Apgar) scores; NICU admissions; mortality rates; and macrosomia. Exclusion criteria involve studies not centered on pregnant women, research not addressing obesity prevalence, studies lacking specific maternal or neonatal health outcomes, and articles not published in peer-reviewed journals.

Search Strategy

We conducted a comprehensive search on PubMed, Scopus, and ScienceDirect to determine the prevalence of obesity among pregnant women and assess its impact on maternal and neonatal outcomes. The objective was to investigate the relationship between pregnancy and obesity, focusing on maternal outcomes such as gestational diabetes, preeclampsia, cesarean section rates, and other complications, as well as neonatal outcomes including birth weight, preterm birth, NICU admissions, infant health, and mortality rates. Our search strategy utilized keywords including (“pregnant women” OR “Pregnancy”) AND (“Obesity” OR “overweight” OR “Body mass index” OR “BMI”) AND (“maternal outcomes” OR “gestational diabetes” OR “Preeclampsia” OR “cesarean section” OR “Complications” OR “neonatal outcomes” OR “birth weight” OR “preterm birth” OR “NICU admission” OR “Infant health”) AND (“prevalence” OR “Incidence” OR “Frequency” OR “rate”) NOT (“review”), excluding reviews to ensure data relevance and reliability.

Screening and Extraction

We excluded articles with irrelevant titles from consideration. In the subsequent phase, both the full text and abstracts of the papers were meticulously reviewed to determine their compliance with the inclusion criteria. To streamline the process, titles and abstracts were organized, assessed, and scrutinized for any duplicate entries using reference management software (Endnote X8; St. Helier, Jersey: Clarivate PLC). To ensure the highest quality of selection, a dual screening approach was adopted, involving one screening for the evaluation of titles and abstracts, and another for the comprehensive examination of the entire texts. Once all relevant articles were identified, a structured extraction sheet was created to capture pertinent information aligned with our specific objectives.

Two separate researchers conducted the data extraction process independently. The gathered information included various study attributes like the author's name, publication year, country of origin, study design, sample size, duration of follow-up, and sources of funding. Additionally, details regarding participants, such as age, gender, and nationality, were also collected.

Quality Assessment

In our systematic review, we employed the Newcastle-Ottawa Scale (NOS) as a critical tool for assessing the quality of non-randomized studies included in our analysis. The NOS is widely recognized for its utility in evaluating the methodological quality and risk of bias in observational studies, including cohort and case-control studies. It provides a structured framework for evaluating key aspects of study design, including selection of study groups, comparability, and ascertainment of outcomes. By using the NOS, we were able to systematically appraise the included studies and ensure that only high-quality evidence contributed to our analysis, thereby enhancing the robustness and reliability of our findings.

Statistical Analysis

The statistical analysis was conducted using R Studio (Boston, MA: Posit), employing a proportional meta-analysis to determine the combined prevalence rate of obesity across studies. Relevant data, including prevalence rates and sample sizes, were extracted and prepared. The meta and metafor packages were used to perform the analysis. A random effects model was applied to account for significant heterogeneity among studies, resulting in a pooled prevalence estimate. Additionally, a forest plot was created to visually represent the individual study estimates and the overall pooled prevalence.

Results

Search Results

**Figure 1:**
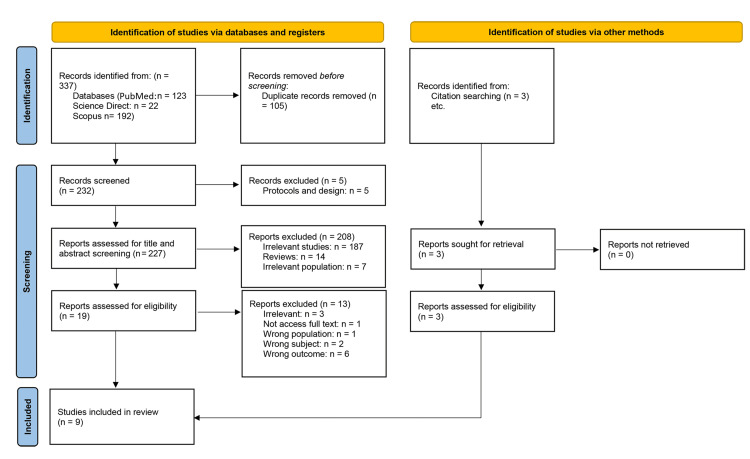
Preferred Reporting Items for Systematic Reviews and Meta-Analyses (PRISMA) flowchart.

We executed the search methodologies outlined previously, resulting in the identification of a total of 340 citations, subsequently reduced to 235 following the removal of duplicates. Upon screening titles and abstracts, only 22 citations met the eligibility criteria for further consideration. Through full-text screening, this number was further refined to nine articles aligning with our inclusion and exclusion criteria [11-19]. Figure 1 provides an in-depth depiction of the search strategy and screening process.

Results of Quality Assessment

The NOS results for the studies assessed indicate a range of quality levels. Studies by Blomberg, Hirooka-Nakama et al., Melchor et al., Alves et al., and Fallatah et al. scored between 7 and 9, denoting "good" to "very good" quality [11,13-16]. These studies demonstrated strong selection and outcome criteria, with adequate to excellent comparability, suggesting minimal risk of bias and high reliability of results. In contrast, studies by Avci et al., Taoudi et al., Adwani et al., and AlAnnaz et al. scored 5, categorizing them as "satisfactory" [12,17-19]. These studies generally had good outcome assessments but lacked comparability and had average selection criteria, indicating the presence of some biases. Overall, while the higher-scoring studies provide more robust and reliable findings, the lower-scoring ones should be interpreted with caution due to potential biases (Table 1).

**Table 1:** Quality assessment of Newcastle-Ottawa Scale (NOS) for included studies.

Study	Selection	Comparability	Outcome	Total score	Quality
Blomberg [11]	3	2	3	8	Good
Avci et al. [12]	2	0	3	5	Satisfactory
Hirooka-Nakama et al. [13]	3	2	3	8	Good
Alves et al. [14]	4	2	3	9	Very good
Melchor et al. [15]	3	2	2	7	Good
Fallatah et al. [16]	4	2	3	9	Very good
Taoudi et al. [17]	3	0	2	5	Satisfactory
Adwani et al. [18]	3	0	2	5	Satisfactory
AlAnnaz et al. [19]	3	0	2	5	Satisfactory

Characteristics of the Included Studies

The included studies, conducted across various countries and published between 2011 and 2024, showcase diverse study designs and periods. Blomberg's cohort study in Sweden (1993-2008) involved 46,595 participants with obesity prevalence categorized into three classes (class 1: 70.8%, class 2: 21.6%, class 3: 7.6%) [11]. Avci et al.'s cohort study in Turkey (2012-2013) included 931 participants, with 11.06% prevalence of obesity and a mean age of 30.46 years [12]. Hirooka-Nakama et al.'s 2018 retrospective study in Japan, based on data from 2013, covered 6,781 participants with a 27.13% obesity prevalence and a mean age of 32 years, reporting a gestational age of 38.9 weeks [13]. Alves et al.'s retrospective case-control study in Portugal (2013-2016) included 6,582 participants with 13.6% obesity prevalence and a mean age of 31.5 years [14]. Melchor et al.'s cohort study in Spain (2013-2017) involved 11,985 participants, showing 13.3% obesity prevalence, a mean age of 34.05 years, and a gestational age of 39.11 weeks [15]. Fallatah et al.'s retrospective study in Saudi Arabia (2013-2018) included 1,037 participants with a mean age of 31.96 years [16]. Taoudi et al.'s cross-sectional study in Morocco (2018-2019) involved 390 participants with a 41% obesity prevalence and a gestational age of 39.53 weeks [17]. Adwani et al.'s retrospective cross-sectional study in Saudi Arabia involved 186 participants with a mean age of 31.94 years [18]. Lastly, AlAnnaz et al.'s 2024 retrospective cross-sectional study in Saudi Arabia included 341 participants with 40.54% obesity prevalence and a mean age of 30.5 years (Table 2) [19].

**Table 2:** Baseline characteristics of included studies. RCT: randomized controlled trial; NR: not reported (obese population only)

Author	Country	Year	Study design	Study period	Total participants	Prevalence of obesity	Mean age of obesity (years)	Gestational age (weeks)
Blomberg [11]	Sweden	2011	Cohort	1993-2008	46,595	Class 1: 70.8%, class 2: 21.6%, class 3: 7.6%	NR	NR
Avci et al. [12]	Turkey	2015	Cohort	2012-2013	931	11.06%	30.46±6.67	NR
Hirooka-Nakama et al. [13]	Japan	2018	Retrospective	2013	6,781	27.13%	32.0±5.3	38.9
Alves et al. [14]	Portugal	2019	Retrospective case-control	2013-2016	6,582	13.6%	31.5±5.5	NR
Melchor et al. [15]	Spain	2019	Cohort	2013-2017	11,985	13.3%	34.05±4.94	39.11
Fallatah et al. [16]	Saudi Arabia	2019	Retrospective	2013-2018	1,037	-	31.96±5.79	NR
Taoudi et al. [17]	Morocco	2021	Cross-sectional	2018-2019	390	41%	NR	39.53
Adwani et al. [18]	Saudi Arabia	2021	Retrospective cross-sectional	2018	186	-	31.94±5.67	NR
AlAnnaz et al. [19]	Saudi Arabia	2024	Retrospective cross-sectional	2021	341	40.54%	30.5±5.24	NR

Study outcome measures

Association of Obesity and Maternal Outcomes in Included Studies

The studies reviewed highlight a strong association between maternal obesity and increased rates of cesarean delivery. Across various obesity classes, higher GWG is consistently linked to elevated odds of cesarean section, with the risk notably increasing in more severe obesity cases. While several studies demonstrate a statistically significant relationship between obesity and cesarean rates, particularly among women with extreme obesity and substantial GWG, others suggest that lower weight gain may help mitigate this risk. Additionally, the presence of maternal comorbidities, such as pre-existing thyroid disease, further elevates cesarean rates among obese women. Overall, these findings emphasize the critical need for managing weight gain during pregnancy to reduce the risk of cesarean delivery in obese patients (Table 3).

**Table 3:** Association of obesity with cesarean delivery and maternal outcomes in included studies. aOR: adjusted odds ratio; CI: confidence interval; GWG: gestational weight gain; OR: odds ratio

Author	Method	Outcome	Details	p-Value
Blomberg [11]	Chi-square (Mantel-Haenszel technique used adjusted OR)	Cesarean section: obese class 1	Weight gain <0 kg, OR: 0.76, 95% CI: 0.65-0.89	-
			Weight gain = 0-4.9 kg, OR: 0.89, 95% CI: 0.80-0.99	-
			Weight gain = 5-9 kg, OR: 1.00, 95% CI: 0.80-1.00	-
			Weight gain >9 kg, OR: 1.23, 95% CI: 1.16-1.31	-
		Obese class 2	Weight gain <0 kg, OR: 0.66, 95% CI: 0.54-0.82	-
			Weight gain = 0-4.9 kg, OR: 0.87, 95% CI: 0.74-1.01	-
			Weight gain = 5-9 kg, OR: 1.00, 95% CI: 0.74-1.01	-
			Weight gain >9 kg, OR: 1.17, 95% CI: 1.05-1.31	-
		Obese class 3	Weight gain <0 kg, OR: 0.77, 95% CI: 0.60-0.99	-
			Weight gain = 0-4.9 kg, OR: 0.82, 95% CI: 0.65-1.04	-
			Weight gain = 5-9 kg, OR: 1.00, 95% CI: 0.84-1.51	-
			Weight gain >9 kg, OR: 1.12, 95% CI: 0.94-1.35	-
Avci et al. [12]	Chi-square	Cesarean section	40.8%	<0.01
Hirooka-Nakama et al. [13]	Logistic regression	Cesarean delivery	GWG <0 kg, OR: 0.86, 95% CI: 0.61-1.22	0.39
			Weight gain 0 kg ≤GWG <5 kg, OR: 1.06, 95% CI: 0.82-1.38	0.91
			Weight gain 9 kg ≤GWG, OR: 1.13, 95% CI: 0.84-1.51	0.41
Alves et al. [14]	Logistic regression	Cesarean section	Obesity class 1, aOR: 1.78, 95% CI: 1.41-2.25	-
			Obesity class 2, aOR: 2.61, 95% CI: 1.77-3.8	-
			Obesity class 3, aOR: 3.19, 95% CI: 1.79-5.71	-
Melchor et al. [15]	Multivariate logistic regression analyses	Cesarean section	aOR: 2.755, 95% CI: 2.46-3.08	<0.000
Taoudi et al. [17]	Pearson's chi-square test or Fisher's exact test	Cesarean section	53.8%	0.018
Adwani et al. [18]	Fisher’s exact test	Pre-existing thyroid disease: Obese class 1	87.5%	0.015
		Obese class 2	100%	-
		Obese class 3	100%	-
AlAnnaz et al. [19]	Chi-square, bivariate correlation (Pearson’s test)	Cesarean section	Chi-square: 5.466	0.050

Association of Obesity With Preeclampsia and Maternal Outcomes Across Studies

The studies indicate a clear association between higher weight gain during pregnancy and an increased risk of preeclampsia in obese women, particularly in higher obesity classes. However, some studies report no significant association, highlighting the complexity of the relationship between obesity, gestational weight gain, and preeclampsia. Overall, the evidence suggests that managing weight gain may reduce the risk of preeclampsia in obese pregnant women (Table 4).

**Table 4:** Association between obesity and preeclampsia in maternal outcomes across studies. aOR: adjusted odds ratio; CI: confidence interval; GWG: gestational weight gain; OR: odds ratio

Author	Method	Outcome	Details	p-Value
Blomberg [11]	Chi-square (Mantel-Haenszel technique used adjusted OR)	Preeclampsia: obese class 1	Weight gain <0 kg, OR: 0.73, 95% CI: 0.54-1.00	-
			Weight gain = 0-4.9 kg, OR: 0.90, 95% CI: 0.74-1.10	-
			Weight gain = 5-9 kg, OR: 1.00, 95% CI: 0.80-1.00	-
			Weight gain >9 kg, OR: 1.66, 95% CI: 1.49-1.85	-
		Obese class 2	Weight gain <0 kg, OR: 1.01, 95% CI: 0.74-1.39	-
			Weight gain = 0-4.9 kg, OR: 0.78, 95% CI: 0.60-1.01	-
			Weight gain = 5-9 kg, OR: 1.00, 95% CI: 0.74-1.01	-
			Weight gain >9 kg- OR: 1.58, 95% CI: 1.28-1.77	-
		Obese class 3	Weight gain <0 kg- OR: 0.74, 95% CI: 0.51-1.08	-
			Weight gain = 0-4.9 kg, OR: 0.65, 95% CI: 0.45-0.94	-
			Weight gain = 5-9 kg, OR: 1.00, 95% CI: 0.84-1.51	-
			Weight gain >9 kg, OR: 1.14, 95% CI: 0.89-1.46	-
Hirooka-Nakama et al. [13]	Logistic regression	Preeclampsia	GWG <0 kg, OR: 0.92, 95% CI: 0.32-2.60	0.87
			Weight gain 0 kg ≤GWG <5 kg, OR: 0.86, 95% CI: 0.55-1.35	0.52
			Weight gain 9 kg ≤GWG, OR: 1.94, 95% CI: 1.31-2.85	<0.001
Melchor et al. [15]	Multivariate logistic regression analyses	Preeclampsia	aOR: 2.199, 95% CI: 1.46-3.29	<0.000
Fallatah et al. [16]	Binary and multinomial logistic regression	Mild preeclampsia	1 (0.91-1.09)	-
		Severe preeclampsia	1 (0.88-1.08)	-
AlAnnaz et al. [19]	Chi-square, bivariate correlation (Pearson’s test)	Preeclampsia	Chi-square: 1.129	0.569

Association of Obesity With Various Maternal Outcomes in Included Studies

The analysis of maternal outcomes related to obesity reveals significant associations across various conditions. Obesity is strongly linked to an increased prevalence of GDM and hypertension (HT), with reported percentages of 14.6% and 2.9%, respectively. Additionally, the risk of hypertensive pregnancy disorders is elevated across all obesity classes, with adjusted odds ratios (aORs) reaching up to 6.38 for the most severe obesity class. The likelihood of developing gestational diabetes also rises with increasing obesity, with aOR values ranging from 1.98 to 2.42 for different obesity classes. Obesity is associated with higher risks of operative vaginal delivery, maternal rectovaginal Group B Streptococcus positive culture, induction of labor, and meconium-stained amniotic fluid. However, conditions such as postpartum hemorrhage (PPH) and endometritis show minimal association with obesity, as indicated by odds ratios close to 1. These findings highlight the extensive impact of maternal obesity on various adverse outcomes during pregnancy and delivery, underscoring the importance of targeted interventions and careful monitoring for obese pregnant women (Table 5).

**Table 5:** Association of obesity with various maternal outcomes: analysis from included studies. aOR: adjusted odds ratio; CI: confidence interval; GWG: gestational weight gain; OR: odds ratio

Author	Method	Outcome	Details	p-Value
Blomberg [11]	Chi-square (Mantel-Haenszel technique)	Bleeding more than 1,000 mL - obese class 1	Weight gain <0 kg, OR: 0.86, 95% CI: 0.66-1.13	-
			Weight gain = 0-4.9 kg, OR: 1.00, 95% CI: 0.83-1.20	-
			Weight gain = 5-9 kg, OR: 1	-
			Weight gain >9 kg, OR: 1.08, 95% CI: 0.97-1.21	-
		Bleeding more than 1,000 mL - obese class 2	Weight gain <0 kg, OR: 1.18, 95% CI: 0.82-1.69	-
			Weight gain = 0-4.9 kg, OR: 1.09, 95% CI: 0.81-1.48	-
			Weight gain = 5-9 kg, OR: 1	-
			Weight gain >9 kg, OR: 1.31, 95% CI: 1.06-1.63	-
		Bleeding more than 1,000 mL - obese class 3	Weight gain <0 kg, OR: 0.95, 95% CI: 0.57-1.59	-
			Weight gain = 0-4.9 kg, OR: 0.71, 95% CI: 0.42-1.19	-
			Weight gain = 5-9 kg, OR: 1	-
			Weight gain >9 kg, OR: 1.09, 95% CI: 0.75-1.59	-
Avci et al. [12]	Chi-square	Gestational diabetes (GDM)	14.6%	<0.01
		Chronic hypertension (HT)	2.9%	<0.01
		Third-trimester hemorrhage	2.90%	0.036
Hirooka-Nakama et al. [13]	Logistic regression	Gestational hypertension	GWG <0 kg, OR: 0.91, 95% CI: 0.59-1.40	0.67
			Weight gain 0 kg ≤GWG <5 kg, OR: 0.85, 95% CI: 0.60-1.19	0.34
			Weight gain 9 kg ≤GWG, OR: 1.14, 95% CI: 0.79-1.63	0.48
		Operative vaginal delivery	GWG <0 kg, OR: 1.75, 95% CI: 1.05-2.94	0.03
			Weight gain 0 kg ≤GWG <5 kg, OR: 0.99, 95% CI: 0.86-1.14	0.67
			Weight gain 9 kg ≤GWG, OR: 1.06, 95% CI: 0.92-1.23	0.41
Alves et al. [14]	Logistic regression	Gestational diabetes	Obesity class 1, aOR: 1.98, 95% CI: 1.35-2.9	-
			Obesity class 2, aOR: 2.42, 95% CI: 1.37-4.26	-
			Obesity class 3, aOR: 2.1, 95% CI: 0.92-4.80	-
		Hypertensive pregnancy disorders	Obesity class 1, aOR: 3.52, 95% CI: 2.27-5.45	-
			Obesity class 2, aOR: 2.54, 95% CI: 1.10-5.85	-
			Obesity class 3, aOR: 6.38, 95% CI: 2.49-16.35	-
Melchor et al. [15]	Multivariate logistic regression analyses	Gestational diabetes	aOR: 0.951, 95% CI: 0.77-1.17	0.637
		Maternal rectovaginal Group B Streptococcus positive culture	aOR: 1.299, 95% CI: 1.14-1.47	<0.000
		Induction of labour	aOR: 1.593, 95% CI: 1.44-1.75	<0.000
		Meconium-stained amniotic fluid	aOR: 1.352, 95% CI: 1.19-1.52	<0.000
Fallatah et al. [16]	Binary and multinomial logistic regression	Vaginal laceration	1.13 (1.04-1.23)	<0.05
		Postpartum hemorrhage (PPH)	1.03 (0.90-1.18)	-
		Endometritis	1.01 (0.85-1.21)	-
		Wound dehiscence	-0.76 (0.23-2.53)	-
AlAnnaz et al. [19]	Chi-square, bivariate correlation (Pearson’s test)	Gestational diabetes	Chi-square: 15.484, p=0.000	<0.000
		Anemia	Chi-square: 6.058, p=0.048	0.048
		Postpartum complications	Chi-square: 17.819, p=0.000	<0.000
		Reason for cesarean section	Chi-square: 27.202, p=0.039	0.039
		Types of postpartum complications	Chi-square: 30.655, p=0.001	<0.001
		Maternal length of stay in hospital	Chi-square: 3.289, p=0.039	0.039

Association Between Obesity and Large for Gestational Age (LGA) Neonatal Outcomes in Included Studies

The data presented in Table 6 illustrates a significant association between maternal obesity and the incidence of LGA in neonates. The studies reveal that in all obesity classes, increased GWG correlates with higher odds of LGA. Specifically, higher weight gain (>9 kg) significantly raises the risk of LGA, particularly in obese classes 1, 2, and 3, with aOR ranging from 1.62 to 7.00. Conversely, lower weight gain is generally associated with reduced odds of LGA. These findings emphasize the impact of maternal obesity and weight gain on neonatal outcomes, highlighting the need for careful management of weight gain during pregnancy to mitigate the risk of LGA.

**Table 6:** Association between obesity and LGA neonatal outcomes in included studies. aOR: adjusted odds ratio; CI: confidence interval; LGA: large for gestational age; OR: odds ratio

Author	Method	Outcome	Details	p-Value
Blomberg [11]	Chi-square (Mantel-Haenszel)	LGA - obese class 1, weight gain <0	aOR: 0.73, 95% CI: 0.58-0.92	-
		LGA - obese class 1, weight gain 0-4.9	aOR: 0.81, 95% CI: 0.69-0.95	-
		LGA - obese class 1, weight gain >9	aOR: 1.96, 95% CI: 1.80-2.14	-
		LGA - obese class 2, weight gain <0	aOR: 0.54, 95% CI: 0.40-0.72	-
		LGA - obese class 2, weight gain 0-4.9	aOR: 0.82, 95% CI: 0.65-1.04	-
		LGA - obese class 2, weight gain >9	aOR: 0.77, 95% CI: 0.63-0.95	-
		LGA - obese class 3, weight gain <0	aOR: 0.64, 95% CI: 0.46-0.90	-
		LGA - obese class 3, weight gain 0-4.9	aOR: 0.87, 95% CI: 0.65-1.17	-
		LGA - obese class 3, weight gain >9	aOR: 1.62, 95% CI: 1.29-2.03	-
Avci et al. [12]	Chi-square	LGA	-	<0.01
Hirooka-Nakama et al. [13]	Logistic regression	LGA - GWG <0 kg	OR: 0.45, 95% CI: 0.30-0.67	<0.01
		LGA - 0 kg ≤GWG < 5 kg	OR: 0.71, 95% CI: 0.54-0.93	0.013
		LGA - 9 kg ≤GWG	OR: 1.43, 95% CI: 1.09-1.88	0.011
Alves et al. [14]	Logistic regression	LGA - obese class 1	aOR: 1.69, 95% CI: 1.17-2.44	-
		LGA - obese class 2	aOR: 3.93, 95% CI: 2.36-6.60	-
		LGA - obese class 3	aOR: 7.0, 95% CI: 3.42-14.30	-

Association Between Obesity and Small for Gestational Age (SGA) Neonatal Outcomes in Included Studies

The analysis of the studies reveals a nuanced relationship between maternal obesity and the likelihood of SGA in neonates. In obese class 1, lower gestational weight gain (<0 kg) significantly increases the odds of SGA (aOR: 2.14), while higher weight gain (>9 kg) is associated with a reduced risk (aOR: 0.54). Similarly, in obese class 2, lower weight gain correlates with a higher risk of SGA (aOR: 1.01), but higher weight gain is linked to a decreased risk (aOR: 0.64). For obese class 3, lower weight gain notably increases the risk of SGA (aOR: 2.34), though higher weight gain does not show a clear pattern. These findings highlight that inadequate gestational weight gain is a more consistent risk factor for SGA among obese women, whereas the impact of higher weight gain varies across different obesity classes (Table 7).

**Table 7:** Association between obesity and SGA neonatal outcomes in included studies. aOR: adjusted odds ratio; CI: confidence interval; GWG: gestational weight gain; SGA: small for gestational age; OR: odds ratio

Author	Method	Outcome	Details	p-Value
Blomberg [11]	Chi-square (Mantel-Haenszel)	SGA - obese class 1, weight gain <0	aOR: 2.14, 95% CI: 1.56-2.95	-
		SGA - obese class 1, weight gain 0-4.9	aOR: 1.62, 95% CI: 1.23-2.11	-
		SGA - obese class 1, weight gain >9	aOR: 0.54, 95% CI: 0.49-0.66	-
		SGA - obese class 2, weight gain <0	aOR: 1.01, 95% CI: 0.54-1.90	-
		SGA - obese class 2, weight gain 0-4.9	aOR: 1.55, 95% CI: 1.03-2.35	-
		SGA - obese class 2, weight gain >9	aOR: 0.64, 95% CI: 0.45-0.91	-
		SGA - obese class 3, weight gain <0	aOR: 2.34, 95% CI: 1.15-4.76	-
		SGA - obese class 3, weight gain 0-4.9	aOR: 1.58, 95% CI: 0.75-3.33	-
		SGA - obese class 3, weight gain >9	aOR: 0.94, 95% CI: 0.50-1.75	-
Avci et al. [12]	Chi-square	SGA	-	<0.01
Hirooka-Nakama et al. [13]	Logistic regression	SGA - GWG <0 kg	OR: 1.44, 95% CI: 0.74-2.82	0.29
		SGA - 0 kg ≤GWG <5 kg	OR: 1.24, 95% CI: 0.68-2.23	0.48
		SGA - 9 kg ≤GWG	OR: 0.96, 95% CI: 0.55-1.69	0.89
Alves et al. [14]	Logistic regression	SGA - obese class 1	aOR: 0.59, 95% CI: 0.41-0.84	-
		SGA - obese class 2	aOR: 0.62, 95% CI: 0.35-1.10	-
		SGA - obese class 3	aOR: 0.1, 95% CI: 0.02-0.50	-

Association Between Obesity and Macrosomia Neonatal Outcomes in Included Studies

The studies indicate a clear association between maternal obesity and an increased risk of macrosomia in neonates. Obesity, particularly in higher classes, is significantly linked to a higher incidence of macrosomia. For instance, in obese class 1, the adjusted odds ratio (aOR) for macrosomia is 2.25, which rises substantially to 5.02 in obese class 2 and 9.53 in obese class 3. This trend reflects a progressively higher risk associated with more severe obesity. Additionally, gestational weight gain (GWG) also plays a crucial role; while low GWG (<0 kg) is associated with a decreased risk of macrosomia, higher GWG (≥9 kg) notably increases the risk, with an odds ratio of 2.09. These findings underscore the significant impact of both maternal obesity and gestational weight gain on the likelihood of delivering a macrosomic infant (Table 8).

**Table 8:** Association between obesity and macrosomia neonatal outcomes in included studies. aOR: adjusted odds ratio; CI: confidence interval; GWG: gestational weight gain; OR: odds ratio

Author	Method	Outcome	Details	p-Value
Hirooka-Nakama et al. [13]	Logistic regression	Macrosomia	GWG <0 kg, OR: 0.23 (0.05-1.01)	0.05
			Weight gain 0 kg ≤GWG <5 kg, OR: 0.76 (0.38-1.50)	0.43
			Weight gain 9 kg ≤GWG, OR: 2.09 (1.08-4.07)	0.03
Alves et al. [14]	Logistic regression	Macrosomia	Obese class 1, aOR: 2.25 (1.35-3.74)	-
			Obese class 2, aOR: 5.02 (2.47-10.20)	-
			Obese class 3, aOR: 9.53 (3.70-24.60)	-
Melchor et al. [15]	Multivariate logistic regression	Macrosomia	≥4,000 g, aOR: 2.090 (1.80-2.42)	<0.000
			≥4,500 g, aOR: 3.087 (2.18-4.37)	<0.000
Fallatah et al. [16]	Binary and multinomial logistic regression	Macrosomia	OR: 1 (0.63-1.49)	<0.001

Association of Obesity With Neonatal Outcomes in Included Studies

Obesity impacts several key neonatal outcomes, including Apgar scores, birth weight, and the need for neonatal intensive care unit (NICU) admission. For instance, maternal obesity, particularly in higher classes, is linked to an increased risk of low Apgar scores at 5 min and fetal distress. However, the results are inconsistent, with some studies reporting elevated adjusted odds ratios (aOR) for adverse outcomes such as NICU admission and low birth weight, while others show minimal impact. Notably, the risk of perinatal death and admission to the NICU is significantly higher in obese mothers, with adjusted odds ratios of 1.34 for NICU admission and 1.33 for umbilical cord arterial pH <7.10. Despite some studies indicating increased risks, other outcomes like low birth weight and neonatal mortality present mixed findings. Overall, these results underscore the complex relationship between maternal obesity and neonatal outcomes, highlighting the need for continued research and targeted interventions to mitigate risks associated with obesity during pregnancy (Table 9).

**Table 9:** Association of obesity with neonatal outcomes: analysis of included studies. aOR: adjusted odds ratio; CI: confidence interval; GWG: gestational weight gain; OR: odds ratio; LBW: low birth weight; IUFD: intrauterine fetal demise

Author	Method	Outcome	Details (OR, CI, and any relevant values)	p-Value
Blomberg [11]	Chi-square (Mantel-Haenszel)	Apgar score <7 at 5 min	Obese class 1: weight gain <0 (2.0%, aOR: 1.23, 95% CI: 0.81-1.86)	-
			Obese class 1: weight gain = 0-4.9 (1.4%, aOR: 0.85, 95% CI: 0.61-1.20)	-
			Obese class 1: weight gain >9 (1.6%, aOR: 0.91, 95% CI: 0.74-1.11)	-
			Obese class 2: weight gain <0 (1.7%, aOR: 0.99, 95% CI: 0.52-1.89)	-
			Obese class 2: weight gain = 0-4.9 (2.8%, aOR: 1.68, 95% CI: 1.10-2.55)	-
			Obese class 2: weight gain >9 (2.0%, aOR: 1.09, 95% CI: 0.77-1.55)	-
			Obese class 3: weight gain <0 (2.8%, aOR: 1.05, 95% CI: 0.55-2.01)	-
			Obese class 3: weight gain = 0-4.9 (2.3%, aOR: 0.95, 95% CI: 0.49-1.83)	-
			Obese class 3: weight gain >9 (4.2%, aOR: 1.53, 95% CI: 0.93-2.51)	-
		Fetal distress	Obese class 1: weight gain <0 (7.3%, aOR: 1.84, 95% CI: 0.83-1.30)	-
			Obese class 1: weight gain = 0-4.9 (6.7%, aOR: 0.95, 95% CI: 0.81-1.12)	-
			Obese class 1: weight gain >9 (8.6%, aOR: 1.05, 95% CI: 0.96-1.11)	-
			Obese class 2: weight gain <0 (7.1%, aOR: 1.04, 95% CI: 0.76-1.42)	-
			Obese class 2: weight gain = 0-4.9 (8.1%, aOR: 1.16, 95% CI: 0.91-1.47)	-
			Obese class 2: weight gain >9 (9.7%, aOR: 1.20, 95% CI: 1.01-1.42)	-
			Obese class 3: weight gain <0 (6.3%, aOR: 0.68, 95% CI: 0.44-1.05)	-
			Obese class 3: weight gain = 0-4.9 (7.9%, aOR: 0.87, 95% CI: 0.59-1.28)	-
			Obese class 3: weight gain >9 (11.6%, aOR: 1.14, 95% CI: 0.86-1.50)	-
Avci et al. [12]	Chi-square	Perinatal death	Perinatal death (p = 0.022)	0.022
Hirooka-Nakama et al. [13]	Logistic regression	LBW	GWG <0 kg (9.2%, OR: 2.08, 95% CI: 1.14-3.81)	0.02
			Weight gain 0 kg ≤GWG <5 kg (4.9%, OR: 1.05, 95% CI: 0.60-1.82)	0.87
			Weight gain 9 kg ≤GWG (4.0%, OR: 0.85, 95% CI: 0.48-1.50)	0.58
		5 min Apgar	GWG <0 kg (2.4%, OR: 1.94, 95% CI: 0.65-5.81)	0.24
			Weight gain 0 kg ≤GWG <5 kg (0.9%, OR: 0.69, 95% CI: 0.24-1.99)	0.49
			Weight gain 9 kg ≤GWG (0.4%, OR: 0.35, 95% CI: 0.09-1.42)	0.14
		Umbilical arterial pH <7	Weight gain 0 kg ≤GWG <5 kg (0.4%, OR: 0.65, 95% CI: 0.11-3.92)	0.64
			Weight gain 9 kg ≤GWG (0.4%, OR: 0.82, 95% CI: 0.14-5.00)	0.83
Alves et al. [14]	Logistic regression	Infants with low birth weight	Obese class 1, aOR: 0.71, 95% CI: 0.49-1.03	-
			Obese class 2, aOR: 0.45, 95% CI: 0.24-0.85	-
			Obese class 3, aOR: 0.08, 95% CI: 0.20-0.38	-
		Admission to neonatal intensive care unit	aOR: 1.341, 95% CI: 1.12-1.59	-
		Umbilical cord arterial pH	aOR: 1.330, 95% CI: 1.12-1.56	-
Melchor et al. [15]	Multivariate logistic regression analyses	Low birth weight	aOR: 0.794, 95% CI: 0.65-0.96	0.021
		Admission to neonatal intensive care unit	aOR: 1.341, 95% CI: 1.12-1.59	<0.001
		Umbilical cord arterial pH <7.10	aOR: 1.330, 95% CI: 1.12-1.56	<0.001
		Neonatal mortality (0-28 days)	aOR: 2.205, 95% CI: 0.86-5.62	0.098
Fallatah et al. [16]	Binary and multinomial logistic regression	NICU admission	OR: 1.01, 95% CI: 0.95-1.06	<0.05
		Spina bifida	OR: 1.14, 95% CI: 0.15-9.01	<0.05
		IUFD	OR: 0.94, 95% CI: 0.83-1.07	<0.05
		Neonatal death	OR: 1.01, 95% CI: 0.86-1.17	<0.05
		Low Apgar at 1 min	OR: 0.99, 95% CI: 0.95-1.03	<0.001
		Low Apgar at 5 min	OR: 1.00, 95% CI: 0.96-1.05	<0.001
		Low birth weight	OR: 1.00, 95% CI: 0.67-1.95	<0.001
Taoudi et al. [17]	Pearson chi-square test or Fisher's exact test	Apgar <7	95% CI: 67.2-76.2	-
		Apgar ≥7	95% CI: 23.8-32.8	-
Adwani et al. [18]	Fisher’s exact test	Gestational age	-	0.325
		IUFD	-	0.312
		Preterm baby	-	0.894
		Apgar score	-	0.751
		Neonatal mortality	-	0.312
AlAnnaz et al. [19]	Bivariate correlation (Pearson’s test)	Birth weight	Pearson’s test: 23.865	<0.000
		First-minute Apgar score	Pearson’s test: 3.003	0.041
		Fifth-minute Apgar score	Pearson’s test: 2.034	0.132
		NICU admission	Pearson’s test: 4.689	0.096

Prevalence of Obesity in Included Studies

**Figure 2:**
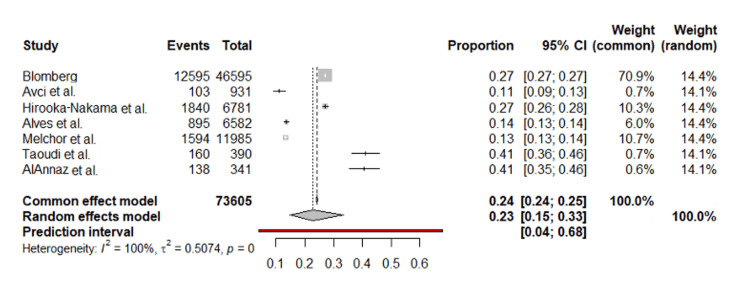
Forest plot of combined obesity prevalence rates across studies.

The meta-analysis of obesity prevalence across seven studies reveals a combined prevalence rate of 22.76% using a random effects model, accounting for the significant variability among the studies. However, the high heterogeneity (I^2^=99.6%) indicates substantial differences in prevalence rates across different populations and study designs. The prediction interval for the random effects model, ranging from 3.99% to 67.63%, further underscores this variability (Figure 2).

Discussion

This systematic review assessed the prevalence of obesity among pregnant women and evaluated its impact on maternal and neonatal outcomes. The pooled incidence of obesity is 22.76%, while the findings highlight significant associations between maternal obesity and adverse outcomes for both mothers and neonates, respectively. Increased GWG in obese individuals correlates with a higher risk of complications such as cesarean delivery, preeclampsia, and postpartum hemorrhage. Specifically, obesity has been consistently linked to higher rates of GDM, which further elevates the likelihood of cesarean sections and other complications during labor. Additionally, in terms of neonatal outcomes, studies reveal that maternal obesity influences the incidence of LGA infants, often leading to macrosomia, while simultaneously being associated with a reduced risk of small for gestational age (SGA) births. Neonates born to obese mothers may also have increased rates of NICU admissions, reflecting the challenges posed by higher maternal weight and its associated risks.

Prevalence of Obesity and Its Effect on Maternal Outcomes

In this review, the meta-analysis of obesity prevalence across seven studies determined an average prevalence rate of 22.76% using a random effects model, taking into account the significant differences observed among the individual studies. Slightly more than our pooled incidence Gaillard reported in their review that the obesity prevalence rate in pregnant women is estimated to be up to 30% [20]. Another meta-analysis from the present time by Kent et al. highlighted that in the most recent full decade, 2010-2019, the global prevalence of maternal obesity was predicted to be 16.3% (95% CI: 15.1-17.5%), or almost one in every six pregnancies. The combined prevalence of overweight/obesity during pregnancy was 43.8% (95% CI: 42.2-45.4%), accounting for nearly half of all pregnancies. Each continent had an upward tendency, similar to the global trend. North America had the highest prevalence (obesity: 18.7% {95% CI: 15.0-23.2%}; overweight/obesity: 47.0% {95% CI: 45.7-48.3%}), while Asia had the lowest prevalence (obesity: 10.8% {95% CI: 7.0-16.5%}; overweight/obesity: 28.5% {95% CI: 18.3-41.5%}). Annually, maternal obesity and combined overweight/obesity prevalence increased by 0.34% and 0.64% (p<0.001), respectively. The linear regression model predicts the present global prevalence of maternal obesity at 20.9% (95% CI: 18.6-23.1%), with a projected increase to 23.3% (95% CI: 20.3-26.2%) by 2030 [21]. The combined prevalence in our review, at 22.76%, slightly exceeds this study’s estimated global prevalence of 20.9% determined through linear regression.

Additionally, our results indicate that obese mothers are more likely to experience complications such as preeclampsia and postpartum hemorrhage due to more substantial GWG, which increases the risk of necessitating a cesarean section and facing additional complications during labor. Also, maternal obesity leads to LGA newborns, which frequently results in macrosomia. Similar to our findings, results of another systematic review of current times highlighted that overweight and obese mothers have a higher likelihood of experiencing cesarean deliveries both elective and emergency; GDM, gestational hypertension, labor induction, postpartum hemorrhage, preeclampsia, and preterm premature rupture of membranes [22]. Likewise, Rahman et al. demonstrated in their meta-analysis findings that overweight or obese mothers are more likely to experience GDM, preeclampsia, cesarean birth, and postpartum hemorrhage compared to those with normal BMI [23]. Findings of a cohort study from Saudi Arabia depicted that GDM was diagnosed in 50.2% of participants, while obesity was identified in 47.8% of them. Obese women with GDM were the oldest and heaviest of all women. Maternal obesity appears to influence birth weight more than GDM; however, GDM is associated with a higher risk of admission to the NICU [24]. The reported incidence of obesity in pregnancy in this study is much higher than our prevalence. While results of a cohort study from Oman showed that obese women had a 2.1 (95% CI: 1.2-3.2) higher risk of cesarean section than normal-weight women, while overweight women had 1.4 (95% CI: 0.9-2.3). The risk of elective cesarean section increased to 7.5 (95% CI: 1.7-32.8) in obese women, which was statistically significant. Obese women had a higher rate of miscarriage (11.9%, n=27) than normal-weight or overweight women (6.7%, 9.4%). There was a minor but statistically significant relationship between birth weight and BMI. Obese women were much more likely to develop macrosomia than normal-weight women [25].

Findings of an Australian retrospective analysis demonstrated that there were 2466 births to women with obesity, including class 1 (69.1%), class 2 (21.8%), and class 3 (9.2%). Almost 42.5% were delivered via cesarean section, 22.3% developed GDM, and 11.2% experienced a hypertensive disorder during pregnancy, with cesarean section and GDM being more likely in women with higher class obesity. LGA occurred in 27.3% of women and SGA in 4.0% of women with all types of obesity. LGA rates were 49% higher in women with class 3 obesity compared to those with class 1 obesity (OR=1.49, CI: 1.06-2.09, p=0.02). The presence of diabetes during the index pregnancy had no significant effect on the probability of neonatal LGA among maternal obesity classes [26]. Onubi et al. reported in their systematic review findings that the prevalence of maternal obesity in Africa ranged from 6.5% to 50.7%, with older and multiparous women being more likely to be obese. Obese mothers had a higher risk of poor labor, child, and maternal outcomes [27]. Our findings align well with the findings from the current literature, and differences in prevalence rates may be attributed to intrinsic characteristics of the study, including disparities in ethnicities and population sizes.

Numerous studies demonstrate significant associations between maternal obesity and various chronic conditions, including hypertension, cancer, cardiovascular disease, and diabetes. Obese women, for instance, are nine times more likely to acquire GDM than normal-weight women of the same age. Obesity, when combined with pre-existing or gestational diabetes, increases the risk of cesarean section and maternal morbidity. Furthermore, women who have had GDM are more likely to develop type 2 diabetes later in life. Furthermore, obesity raises the risk of poor glucose metabolism during pregnancy, resulting in excessive fetal growth, including LGA and macrosomia [28]. Khair et al. further agreed in this context as they described that extensive cohort studies and meta-analyses provide strong evidence that maternal obesity and overweight are related to diverse adverse pregnancy outcomes in both mothers and newborns. There is a substantial increase in maternal complications, including the development of GDM, gestational hypertensive disorders, preeclampsia, and the need for surgical births [29].

Godfrey et al. narrated that along with its immediate implications for pregnancy complications, additional evidence suggests that maternal obesity is an important predictor of health in children during infancy and later adulthood. Observational studies show that maternal obesity increases the offspring's risk of obesity, coronary heart disease, stroke, type 2 diabetes, and asthma. Maternal obesity may potentially cause lower cognitive performance in offspring, as well as an increased chance of neurological disorders such as cerebral palsy. Preliminary findings reveal potential implications for immunological and infectious disease outcomes. Experimental findings indicate the causal effects of maternal obesity on offspring outcomes, which are mediated at least in part by changes in epigenetic processes such as DNA methylation and possibly by abnormalities in the gut microbiome [30]. Although obese women who lose weight before pregnancy have a lower risk of obesity, few controlled intervention trials have been conducted to reverse maternal obesity and investigate the repercussions for the progeny. The long-term effects of maternal obesity may have far-reaching public health implications, highlighting the importance of research into causality, underlying mechanisms, and effective interventions to reverse the obesity epidemic in women of childbearing age and mitigate its consequences for children [30].

Effect on Neonatal Outcomes

Regarding neonatal outcomes among the included studies in this review, we noted that maternal obesity impacts the occurrence of macrosomia and shoulder dystocia. Babies born to obese mothers may experience higher rates of NICU admissions and neonatal mortality, highlighting the complexities associated with maternal overweight and its related risks. Similarly, results from another systematic review showed that infants born to obese mothers are at an elevated risk for admission to the NICU, with low Apgar scores below 7 at 5 min, being LGA, macrosomia, and extreme preterm birth when compared to mothers with a normal BMI [22]. Likewise, another meta-analysis from present times highlighted that women with obesity faced higher risks of LGA and macrosomia compared to those of normal weight, with odds ratios of 1.98 (95% CI: 1.56, 2.52) and 2.93 (95% CI: 1.71, 5.03), respectively. The risk of macrosomia was nearly doubled in obese women with GDM. Additionally, these women had higher birth weights (mean difference of 113 g, 95% CI: 69, 156) and an increased risk of shoulder dystocia (OR: 1.23, 95% CI: 0.85, 1.78). GDM significantly heightened neonatal risks in obese women, leading to a three- to four-fold increase in the likelihood of LGA (OR: 3.22, 95% CI: 2.17, 4.79) and macrosomia (OR: 3.71, 95% CI: 2.76, 4.98), along with higher birth weights (mean difference of 176 g, 95% CI: 89, 263), increased chances of preterm delivery (OR: 1.49, 95% CI: 1.25, 1.77), and a greater risk of shoulder dystocia (OR: 1.99, 95% CI: 1.31, 3.03) compared to normal-weight women [31]. However, in this review, we observed that while there is broad agreement among included studies on the relationship between maternal obesity and certain outcomes, such as the increased prevalence of LGA and the need for cesarean delivery, the diversity in findings regarding specific indicators like Apgar scores, and low birth weight points to the complexity of these relationships. This complexity underscores the necessity for ongoing research to better delineate the implications of maternal obesity on both maternal and neonatal health outcomes. Understanding these dynamics is crucial for developing targeted interventions and improving care for obese pregnant individuals.

Additionally, findings of a meta-analysis by Voerman et al. indicated that higher mother pre-pregnancy BMI and GWG were associated with an increased risk of children being overweight/obese, with the most significant impacts occurring later in life [32]. Another meta-analysis by D'Souza et al. highlighted that almost all adverse pregnancy outcomes were directly associated with maternal BMI. Babies were at increased risk for hypoglycemia, macrosomia, infection, birth trauma, respiratory distress, and death [33].

Adding further evidence, Marchi et al. concluded in their review that maternal obesity has also been associated with an increased risk of preterm birth, LGA newborns, fetal abnormalities, congenital malformations, and perinatal mortality. Moreover, breastfeeding initiation rates are lower, and women with obesity are more likely to discontinue breastfeeding earlier than women of normal weight. These negative outcomes may result in a lengthier hospital stay, which has resource consequences [34]. Similarly, Kureshi et al. concluded in their review findings that maternal obesity has also been associated with an increased risk of preterm birth, LGA newborns, fetal abnormalities, congenital malformations, and perinatal mortality. Furthermore, breastfeeding initiation rates are lower, and women with obesity are more likely to discontinue breastfeeding earlier than women of normal weight. These negative outcomes may result in a lengthier hospital stay, which has resource consequences [35]. Although in this review we did not consider observing the impact of maternal obesity on the initiation of breastfeeding rates, as we considered it beyond the scope and objective of this study.

Catalano and Shankar highlighted that newborns of obese mothers had greater levels of umbilical cord leptin and interleukin-6 than newborns of lean mothers. Furthermore, neonates of obese moms have higher insulin resistance than those of lean mothers. Insulin resistance in obese newborns was found to be highly linked with maternal insulin resistance and neonatal body fat. Placental weight was the best predictor of newborn fat accumulation in both male and female neonates (r^2^=0.20-0.39). However, in male newborns, maternal BMI and GWG were strong predictors of both lean and fat mass. In contrast, maternal plasma inflammatory indicators (IL-6 and C-reactive protein) were independently linked to female body fat and lean body mass [4]. Findings of a cohort study by Gaudet et al. showed that newborns delivered to mothers with class 3 obesity had an increased risk of fetal overgrowth and low cord artery pH. However, class 3 obesity protects against being SGA and having a low birth weight. There was no difference in the risk of preterm labor, meconium in the amniotic fluid, or breastfeeding initiation [36]. Another cohort study by Yao et al. reported that the incidence of stillbirth was 1.4 per 1,000 births among women of normal weight, compared to 2.9 per 1,000 among obese women (p<0.001, aOR: 1.83 {1.43, 2.34}). Neonatal death rates were 4.3 per 1,000 births for normal-weight women and 4.7 per 1,000 for obese women (p=0.94, aOR: 1.10 {0.92, 1.30}). Maternal obesity showed a progressive relationship with stillbirths but not with other neonatal outcomes. Among SGA infants, maternal obesity before pregnancy correlated with higher risks of stillbirth, NICU admission, and low Apgar scores, but not neonatal death [37]. In this review, umbilical arterial pH <7 was discussed in only two of the included studies with one of them reporting a significant association, while congenital anomalies were reported by only one study [13,15,16].

This systematic review offers a valuable synthesis of existing research on how obesity affects pregnant women and their babies. By meticulously analyzing a wide range of studies, the review provides a comprehensive understanding of obesity prevalence in maternal populations and its significant implications for maternal health outcomes, such as gestational diabetes, hypertension, and cesarean deliveries. Moreover, it examines the impact on neonatal outcomes, including the incidence of macrosomia and NICU admissions among infants born to obese mothers. Through rigorous statistical methods and meta-analysis, the review ensures robustness in its findings, offering evidence-based insights that are crucial for clinicians, policymakers, and researchers alike and defining the strength of this study. These insights not only contribute to clinical decision-making but also inform public health strategies aimed at mitigating the adverse effects of maternal obesity, ultimately improving the health outcomes of both mothers and their babies. Moreover, the systematic search methodology and the analysis of all keywords in this field further add to the advantages and strengths of this study.

Limitations and future research directions

However, despite its strengths, this study has several limitations. First, variability in methodologies and definitions of obesity across included studies could introduce heterogeneity, potentially affecting the comparability and generalizability of findings. Additionally, including a majority of satisfactory quality studies may limit the generalizability of findings. The review's reliance on existing literature also means it is limited by the quality and comprehensiveness of available studies, potentially omitting unpublished or less accessible data. Moreover, while the review synthesizes data on associations between maternal obesity and health outcomes, it may not always establish causality due to the observational nature of most included studies. Finally, the review's scope may be limited to certain geographical regions or specific populations, which could restrict the applicability of its findings to diverse global contexts. Addressing these limitations would strengthen the review's reliability and applicability in informing clinical practice and public health strategies related to maternal obesity. Future research directions should focus on elucidating the mechanistic pathways through which maternal obesity contributes to adverse pregnancy outcomes, such as gestational diabetes, hypertension, and increased cesarean delivery rates. Longitudinal studies are crucial to understanding the extended health impacts on both mothers and children beyond the immediate postpartum period. Evaluating the effectiveness of various intervention strategies, including lifestyle modifications, behavioral interventions, and pharmacological approaches, will help identify the most effective methods for managing maternal obesity during pregnancy. Additionally, research should delve into how socioeconomic factors influence the prevalence and consequences of maternal obesity, ensuring interventions are equitable and effective across diverse populations. Studying the developmental outcomes of children born to obese mothers, including neurodevelopment and behavioral health, will provide insights into the broader implications of maternal obesity on child health. Finally, exploring health system-level interventions and preventive strategies aimed at reducing maternal obesity before conception can contribute to improving maternal and neonatal health outcomes on a larger scale.

## Conclusions

This study underscores the substantial impact of obesity on pregnancy outcomes. Our analysis consistently links maternal obesity with adverse maternal outcomes, including GDM, preeclampsia, and increased cesarean delivery rates. Moreover, maternal obesity correlates with higher rates of LGA infants and increased NICU admissions while reducing the likelihood of SGA infants. The variability in findings, particularly regarding outcomes such as Apgar scores, emphasizes the need for further research to unravel the complex relationships between maternal obesity and maternal and neonatal health. Future studies should focus on elucidating mechanisms underlying these associations, developing effective interventions to mitigate risks, and optimizing care for obese pregnant individuals to improve outcomes for both mothers and infants.

## References

[1] Safaei M, Sundararajan EA, Driss M, Boulila W, Shapi'i A (2021). A systematic literature review on obesity: understanding the causes & consequences of obesity and reviewing various machine learning approaches used to predict obesity. Comput Biol Med.

[2] Grieger JA, Hutchesson MJ, Cooray SD (2021). A review of maternal overweight and obesity and its impact on cardiometabolic outcomes during pregnancy and postpartum. Ther Adv Reprod Health.

[3] Chen C, Xu X, Yan Y (2018). Estimated global overweight and obesity burden in pregnant women based on panel data model. PLoS One.

[4] Catalano PM, Shankar K (2017). Obesity and pregnancy: mechanisms of short term and long term adverse consequences for mother and child. BMJ.

[5] Orós M, Siscart J, Perejón D, Serna MC, Godoy P, Salinas-Roca B (2023). Ethnic disparities and obesity risk factors in pregnant women: a retrospective observational cohort study. Nutrients.

[6] Langley-Evans SC, Pearce J, Ellis S (2022). Overweight, obesity and excessive weight gain in pregnancy as risk factors for adverse pregnancy outcomes: a narrative review. J Hum Nutr Diet.

[7] Deputy NP, Dub B, Sharma AJ (2018). Prevalence and trends in prepregnancy normal weight - 48 states, New York City, and District of Columbia, 2011-2015. MMWR Morb Mortal Wkly Rep.

[8] (2020). Statistics on obesity, physical activity and diet (replaced by statistics on public health). https://digital.nhs.uk/data-and-information/publications/statistical/statistics-on-obesity-physical-activity-and-diet?utm_medium=email&utm_source=transaction.

[9] Reed J, Case S, Rijhsinghani A (2023). Maternal obesity: perinatal implications. SAGE Open Med.

[10] Mitanchez D, Chavatte-Palmer P (2018). Review shows that maternal obesity induces serious adverse neonatal effects and is associated with childhood obesity in their offspring. Acta Paediatr.

[11] Blomberg M (2011). Maternal and neonatal outcomes among obese women with weight gain below the new Institute of Medicine recommendations. Obstet Gynecol.

[12] Avci ME, Şanlıkan F, Çelik M, Avci A, Kocaer M, Göçmen A (2015). Effects of maternal obesity on antenatal, perinatal and neonatal outcomes. J Matern Fetal Neonatal Med.

[13] Hirooka-Nakama J, Enomoto K, Sakamaki K, Kurasawa K, Miyagi E, Aoki S (2018). Optimal weight gain in obese and overweight pregnant Japanese women. Endocr J.

[14] Alves P, Malheiro MF, Gomes JC, Ferraz T, Montenegro N (2019). Risks of maternal obesity in pregnancy: a case-control study in a Portuguese obstetrical population. Rev Bras Ginecol Obstet.

[15] Melchor I, Burgos J, Del Campo A, Aiartzaguena A, Gutiérrez J, Melchor JC (2019). Effect of maternal obesity on pregnancy outcomes in women delivering singleton babies: a historical cohort study. J Perinat Med.

[16] Fallatah AM, Babatin HM, Nassibi KM, Banweer MK, Fayoumi MN, Oraif AM (2019). Maternal and neonatal outcomes among obese pregnant women in King Abdulaziz University Hospital: a retrospective single-center medical record review. Med Arch.

[17] Taoudi F, Laamiri FZ, Barich F, Hasswane N, Aguenaou H, Barkat A (2021). Study of the prevalence of obesity and its association with maternal and neonatal characteristics and morbidity profile in a population of Moroccan pregnant women. J Nutr Metab.

[18] Adwani N, Fouly H, Omer T (2021). Assessing the impact of obesity on pregnancy and neonatal outcomes among Saudi women. Nurs Rep.

[19] AlAnnaz WA, Gouda AD, El-Soud FA, Alanazi MR (2024). Obesity prevalence and its impact on maternal and neonatal outcomes among pregnant women: a retrospective cross-sectional study design. Nurs Rep.

[20] Gaillard R (2015). Maternal obesity during pregnancy and cardiovascular development and disease in the offspring. Eur J Epidemiol.

[21] Kent L, McGirr M, Eastwood KA (2024). Global trends in prevalence of maternal overweight and obesity: a systematic review and meta-analysis of routinely collected data retrospective cohorts. Int J Popul Data Sci.

[22] Vats H, Saxena R, Sachdeva MP, Walia GK, Gupta V (2021). Impact of maternal pre-pregnancy body mass index on maternal, fetal and neonatal adverse outcomes in the worldwide populations: a systematic review and meta-analysis. Obes Res Clin Pract.

[23] Rahman MM, Abe SK, Kanda M (2015). Maternal body mass index and risk of birth and maternal health outcomes in low- and middle-income countries: a systematic review and meta-analysis. Obes Rev.

[24] Alfadhli EM (2021). Maternal obesity influences birth weight more than gestational diabetes author. BMC Pregnancy Childbirth.

[25] Al-Hakmani FM, Al-Fadhil FA, Al-Balushi LH (2016). The effect of obesity on pregnancy and its outcome in the population of Oman, Seeb province. Oman Med J.

[26] Neal K, Ullah S, Glastras SJ (2022). Obesity class impacts adverse maternal and neonatal outcomes independent of diabetes. Front Endocrinol (Lausanne).

[27] Onubi OJ, Marais D, Aucott L, Okonofua F, Poobalan AS (2016). Maternal obesity in Africa: a systematic review and meta-analysis. J Public Health (Oxf).

[28] Aguree S, Zhang X, Reddy MB (2023). Combined effect of maternal obesity and diabetes on excessive fetal growth: Pregnancy Risk Assessment Monitoring System (PRAMS), United States, 2012-2015. AJPM Focus.

[29] Khair H, Bataineh MF, Zaręba K (2023). Pregnant women's perception and knowledge of the impact of obesity on prenatal outcomes - a cross-sectional study. Nutrients.

[30] Godfrey KM, Reynolds RM, Prescott SL, Nyirenda M, Jaddoe VW, Eriksson JG, Broekman BF (2017). Influence of maternal obesity on the long-term health of offspring. Lancet Diabetes Endocrinol.

[31] Weir TL, Majumder M, Glastras SJ (2024). A systematic review of the effects of maternal obesity on neonatal outcomes in women with gestational diabetes. Obes Rev.

[32] Voerman E, Santos S, Patro Golab B (2019). Maternal body mass index, gestational weight gain, and the risk of overweight and obesity across childhood: an individual participant data meta-analysis. PLoS Med.

[33] D'Souza R, Horyn I, Pavalagantharajah S, Zaffar N, Jacob CE (2019). Maternal body mass index and pregnancy outcomes: a systematic review and metaanalysis. Am J Obstet Gynecol MFM.

[34] Marchi J, Berg M, Dencker A, Olander EK, Begley C (2015). Risks associated with obesity in pregnancy, for the mother and baby: a systematic review of reviews. Obes Rev.

[35] Kureshi A, Khalak R, Gifford J, Munshi U (2022). Maternal obesity-associated neonatal morbidities in early newborn period. Front Pediatr.

[36] Gaudet L, Tu X, Fell D, El-Chaar D, Wen SW, Walker M (2012). The effect of maternal class III obesity on neonatal outcomes: a retrospective matched cohort study. J Matern Fetal Neonatal Med.

[37] Yao R, Park BY, Caughey AB (2017). The effects of maternal obesity on perinatal outcomes among those born small for gestational age. J Matern Fetal Neonatal Med.

